# The Contribution of *COL4A5* Splicing Variants to the Pathogenesis of X-Linked Alport Syndrome

**DOI:** 10.3389/fmed.2022.841391

**Published:** 2022-02-08

**Authors:** Tomohiko Yamamura, Tomoko Horinouchi, Yuya Aoto, Rachel Lennon, Kandai Nozu

**Affiliations:** ^1^Department of Pediatrics, Kobe University Graduate School of Medicine, Kobe, Japan; ^2^Wellcome Centre for Cell-Matrix Research, Faculty of Biology Medicine and Health, University of Manchester, Manchester, United Kingdom; ^3^Department of Paediatric Nephrology, Royal Manchester Children's Hospital, Manchester University Hospitals NHS Foundation Trust, Manchester Academic Health Science Centre, Manchester, United Kingdom

**Keywords:** alport syndrome, *COL4A5*, splicing, genotype phenotype correlation, minigene

## Abstract

X-linked Alport syndrome (XLAS) is caused by pathogenic variants in *COL4A5* and is characterized by progressive kidney disease, hearing loss, and ocular abnormalities. Recent advances in genetic analysis and further understanding of genotype-phenotype correlations in affected male patients raises the importance of detecting splicing variants in *COL4A5*. Aberrant splicing of *COL4A5* is caused not only by canonical splice site variants but also non-canonical splice site variants such as deep intronic changes or even substitutions in exons. Patients with splicing variants account for ~15% of all cases in XLAS. In addition, it has been shown that there is a significant difference in kidney survival depending on the aberrant splicing patterns of transcripts- in particular in-frame or out-of-frame nucleotide changes in transcripts. Therefore, cDNA analysis of patient mRNA is necessary to determine the impact of splice site variants and to confirm a diagnosis of XLAS and to predict the kidney prognosis. However, it is usually difficult to amplify *COL4A5* transcripts extracted from peripheral blood leukocytes. For these cases, *in vitro* minigene assays or RNA sequence extracted from urine derived cells can confirm aberrant splicing patterns. Moreover, controlling aberrant splicing by nucleic acids or small molecular compounds in genetic diseases are attracting attention as a potential therapeutic strategy. Here, we review the frequency of splicing variants in *COL4A5*, the latest diagnostic strategies, and the prospects for new therapeutic approaches.

## Introduction

Alport syndrome (AS) is an inherited disorder with progressive kidney disease, frequently accompanied by sensorineural hearing loss and specific ocular abnormalities (1–4). AS is caused by defects of in the type IV collagen network, a major structural component of basement membranes in the kidney, inner ear, and eye. Six distinct type IV collagen α-chains (α1–α6) have been identified and are encoded by the genes *COL4A1*–*COL4A6*. Pathogenic variants in the *COL4A5*, which encodes the type IV collagen α5 chain, are known to cause X-linked Alport syndrome (XLAS); XLAS is the most common inherited form of AS with ~80% of all AS patients (5). Our group confirmed this distribution in our cohort of all genetically diagnosed Japanese AS families (*n* = 397) where we found 74% (*n* = 295) had XLAS (6).

Regarding the genotype of XLAS, all types of variant category have been registered as causative variants to clinical genetic databases similar to other human inherited diseases. In addition, it is already known that strong genotype and kidney phenotype correlation exists in affected male patients with XLAS; patients with missense or small in-frame variants show less severe phenotypes compared to patients with truncating variants (e.g., nonsense, a small insertion/deletion leading to a premature stop codon) (7–10). In addition, we recently focused on the difference in transcripts of splicing variants in *COL4A5* based on whether the abnormal transcript has in-frame deletion (the total number of nucleotides is multiple of 3) or out-of-frame deletion (not multiple of 3). According to this analysis, we revealed that male patients with splicing variants leading to in-frame transcripts had less severe phenotypes than those with out of frame transcripts (10, 11).

Recent advances in genetic analysis have enabled comprehensive and efficient screening of multiple genes including *COL4A3, COL4A4*, and *COL4A5* for patients suspected as having Alport syndrome. However, specific variants causing abnormal splicing such as deep intronic variants cannot be detected by (targeted) exome sequencing and a consensus approach for detecting deep intronic variants has not been established (12–15). Moreover, transcript analysis targeting genomic DNA variants, which are suspected to causing aberrant splicing is challenging because of the stability of mRNA, and the extremely low expression level of *COL4A5* transcripts in accessible cells such as peripheral blood leukocytes.

In this review, we provide a comprehensive overview of the investigation and functional analysis of splicing variants in the *COL4A5* gene; including the frequency of these variants, the latest diagnostic strategies, and the prospects for new therapeutic approaches to regulate splicing patterns.

## Splicing Abnormalities and Human Genetic Diseases

**Figure 1:**
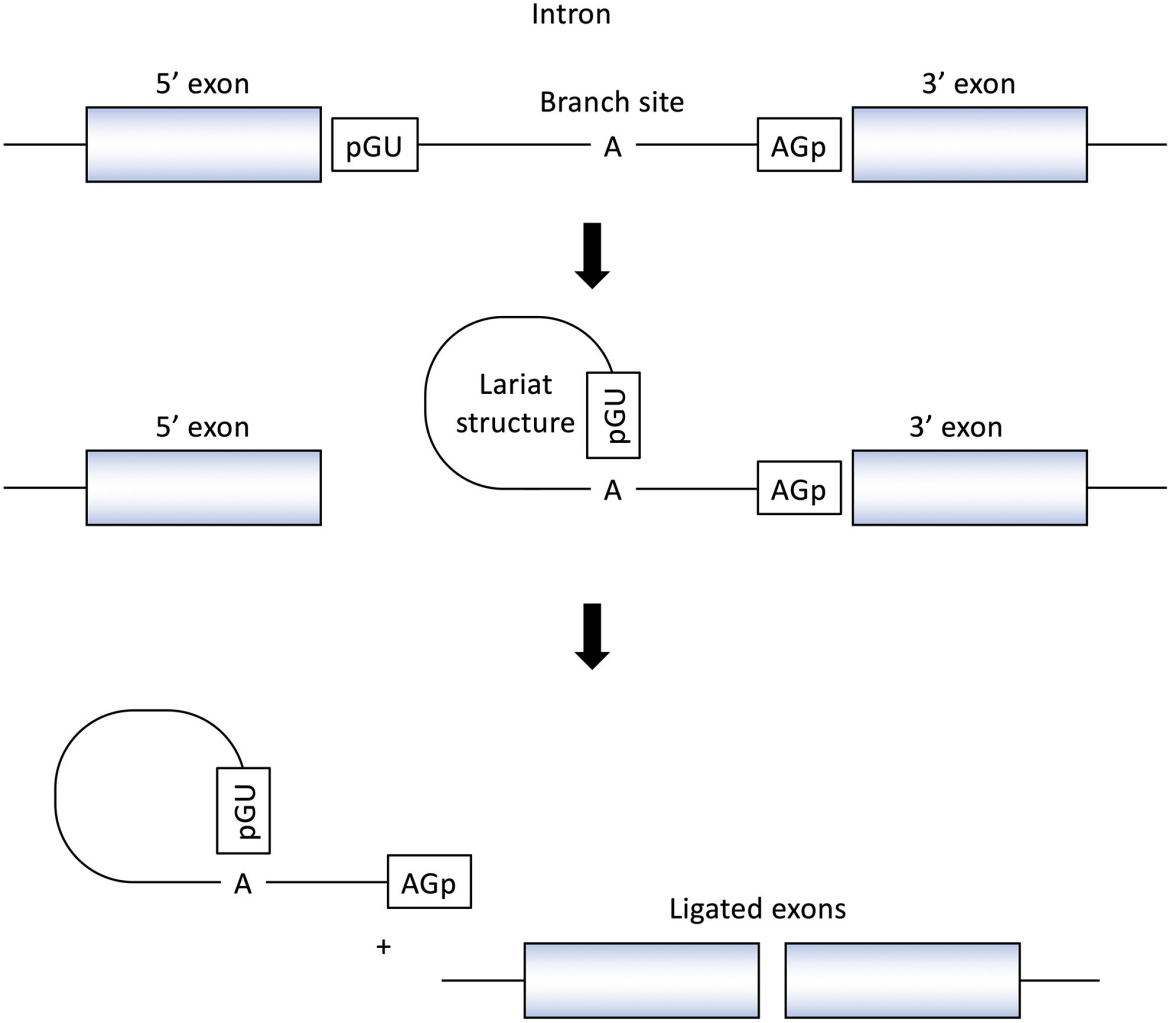
Splicing reactions and important splicing elements. There are several essential splicing motifs located in boundary region between exons and introns. In addition to highly conserved dinucleotides of both 5′ and 3′ side of intron (GU/AG), adenosine (A) at the branch site and polypyrimidine tract (not shown in figure) are located in intron. pre-mRNA splicing process take place in two transesterification steps. In the first step, the 2′-OH group of the adenosine (A) at the branch site performs a nucleophilic attack on a phosphate (p) at the 5′ splice site. This leads to cleavage of the 5′ exon from the intron and the formation of a lariat structure. In the following step, a second transesterification reaction, which involves the phosphate at the 3′ end of the intron, detach the intron from exon and ligates the two exons.

RNA splicing is a form of RNA processing in which precursor messenger RNA (pre-mRNA) is transformed into mature messenger RNA (mRNA) in the sequence of protein biosynthesis. In higher eukaryotes, the nucleotide sequence of genomic DNA (gDNA) is divided into the protein coding region (exon) and non-coding region (intron). Pre-mRNA newly transferred from gDNA include both exons and introns. The process of pre-mRNA splicing removes introns from pre-mRNA, and the remaining exons are combined to form mature mRNA (Figure 1).

In the process of pre-mRNA splicing, specific sequences located in intron play an important role. Dinucleotides of both the 5' and 3' side of intron are highly conserved (GU/AG) and called the splice donor site and the splice acceptor site, respectively. In addition, other conserved motifs such as the branch site and the polypyrimidine tract, located upstream of the 3′ splice site is also important to determine the location of splicing. Pre-mRNA splicing takes place in two transesterification steps. In the first step, the 2′-OH group of the adenosine (A) at the branch site performs a nucleophilic attack on a phosphate (p) at the 5' splice site. This leads to cleavage of the 5′ exon from the intron and the formation of a lariat structure. In the following step, a second transesterification reaction, which involves the phosphate at the 3′ end of the intron, detaches the intron from exon and ligates the two exons (16) (Figure 1).

In addition to above essential splicing motifs, additional sequence elements known as enhancers or silencers are needed for accurate splicing. These regulatory sequences are located both in exons and introns and called exonic/intronic splicing enhancers/silencers. Furthermore, the splicing process, including the precise recognition of the splice site, is catalyzed by the huge complex of proteins and enzymes termed the spliceosome (17).

These splicing motifs or elements are known as a target of variation in genetic diseases. Variants in this region often cause aberrant splicing and result in pathology. The importance of splicing variants is illustrated by the fact that nearly 15% of human genetic diseases are estimated to be caused by variants located in the 5' or 3' consensus splice sites (18). In addition, comprehensive studies of cDNA analysis for all detected pathogenic variants in patients with ataxia-telangiectasia (OMIM#208900) and neurofibromatosis type I (OMIM#162200) revealed that nearly 50% of variants resulted in aberrant splicing patterns (19, 20). Surprisingly, among the splicing variants in these reports, a minority were detected in the conserved dinucleotide of 5' and 3' side (GU/AG) and most of other variants located in exons or other intronic regions and caused abnormal splicing. The mechanism of how variants located in exon region causes aberrant splicing is mainly explained by the disruption of splicing regulatory elements such as exonic splicing enhancer (ESE) or silencer (ESS) (21). If a nucleotide substitution is in the sequence of these important motifs, the signal to be recognized as an exon is weakened and this can cause exon skipping in process of splicing.

## Splicing Variants in *COL4A5* Gene

### Frequency of Splicing Variants in *COL4A5*

To date, several retrospective studies reported the proportion of patients with splicing variants in XLAS. Jais et al. (7) investigated genetic and clinical characteristics in 195 families with XLAS and revealed that 29 families (14.9%) had splice site variants. Bekheirnia et al. (8) also reported the proportion of each variant in 175 families with male XLAS patient and it revealed 13.7% of all families possessed splice site variants. Recently, our group investigated 269 Japanese families with XLAS and we found that splicing variants were detected in 18.2% of all families (Table 1) (10). The higher proportion of splicing variants in our study was thought to be the effect of active transcriptional analysis to detect deep intronic variants and exonic splicing variants other than canonical dinucleotides splice site (GU/AG) variants. Interestingly, among 71 male XLAS patients from 49 families with splicing variants in our study, only 35 patients (26 families) had variants in canonical dinucleotides splice site of *COL4A5* gene. These findings highlight the importance of splicing variants in *COL4A5* and demonstrate similarity to the other inherited diseases mentioned in previous section.

**Table 1:** Mutational characteristics of reported cohort of X-linked Alport syndrome.

	**Jais et al**.		**Bekheirnia et al**.		**Yamamura et al**.	
**Characteristics**	***N* with data**	***n* (%)**	***N* with data**	***n* (%)**	***N* with data**	***n* (%)**
Mutation types in families	195		175		269	
Missense mutation		74 (38.9)		89 (50.9)		144 (53.5)
Nonsense mutation		14 (7.2)		N/A^a^		19 (7.1)
Splicing variant		**29 (14.9)**		**24 (13.7)**		**49 (18.2)**
Small rearrangement		40 (20.5)		N/A^a^		44 (16.4)
Large rearrangement		38 (19.5)		14 (8.0)		13 (4.8)

a *It is unable to specify the numbers of patients with these two types of variants because this study classified nonsense mutations and truncating small rearrangements into “truncating mutation.”*

### Genotype-Phenotype Correlation in Splicing Variants With XLAS

As described above there are strong genotype-phenotype correlations in males with XLAS; in particular, patients with missense or small in-frame variants (so-called non-truncating variants) have less severe phenotypes compared to patients with truncating variants (e.g., nonsense, small insertion, or deletion leading to out-of-frame sequences) (7–9). In addition, patients with splice site variants have shown intermediate severity, between the phenotypes associated with non-truncating and truncating variants.

Although splicing variants can be classified depending on their transcript pattern (i.e., in-frame or out-of-frame transcript), genotype-phenotype correlation analysis based on transcriptional analysis had not been conducted. Recently, we focused on this transcriptional difference in splicing variants and analyzed the kidney survival of patients with splicing variants. We found a significant difference between patients with in-frame splicing variants and those with out-of-frame splicing variants; the median kidney survival of patients with the in-frame splicing variants (*n* = 33) was 28 years, whereas it was 23 years for patients with the out-of-frame splicing variants (*n* = 32; *P* < 0.05) (10). This result demonstrates the value of transcriptional analysis for splicing variants in XLAS to estimate their kidney prognosis and enable genetic counseling.

### Exonic Variants Causing Abnormal Splicing in *COL4A5*

Some exonic variants, which can be considered as missense or nonsense if transcriptional analysis is not conducted, affect splicing and may cause disease. In particular, single-base substitutions at the last nucleotide position in each exon are reported to likely affect splicing patterns (19, 20). However, no studies have addressed the characteristic of exonic variants in the *COL4A5* gene which are likely to affect splicing. Therefore, we focused on the variants affecting the last nucleotide of exons in *COL4A5* and conducted a comprehensive *in vitro* transcript analysis (22). We found 14 reported variants located in last nucleotide of any *COL4A5* exon from the Human Gene Mutation Database (HGMD) and six novel variants from our cohort. All 14 variants in HGMD are reported as missense and most of them are glycine substitution, which is most common type of missense variant in *COL4A5* (3). Furthermore, using an *in vitro* functional splicing analysis, 17 out of the total 20 variants showed aberrant splicing. In this study, we also conducted the genotype-phenotype correlation analysis of the splicing variants caused by substitution of last nucleotide in exons comparing to our previous report of missense variants. We found that the median age of developing end-stage kidney disease (ESKD) in cases with splicing variants was significantly worse than those with missense variants (27 vs. 40 years old, *P* < 0.01). From this result, we concluded that variants located in last nucleotide position of exons in *COL4A5*, even if they are glycine missense substitution, should be considered likely splicing variants and examined by transcriptional analysis.

In addition, the importance of synonymous or silent variants in abnormal splicing in *COL4A5* should be considered. We analyzed *COL4A5* transcripts in three patients clinically diagnosed XLAS with synonymous variants by using the *in vitro* functional splicing assay and analysis of patient mRNA. This revealed that all three cases showed aberrant splicing patterns (23, 24). Interestingly, among these three cases, one patient had both aberrant and normal transcripts by mRNA analysis, and they exhibited a milder phenotype. This finding suggested that synonymous variants in *COL4A5* can affect splicing pattern and might show milder phenotype via producing mixture of both normal transcripts and aberrant splicing.

### Intronic Variants Outside the Canonical Splice Site Causing Abnormal Splicing in *COL4A5*

Although the canonical dinucleotides splice site (GU/AG) is important for correct splicing and variants in this region cause aberrant splicing, intronic variants outside this region may also influence splicing. Indeed, there are several non-canonical intronic variants around exon-intron boundaries that have been reported in genomic databases such as HGMD (14). However, the pathogenicity of those intronic variants were not all proven by transcript analysis. Recently, our group reported the results of transcript analysis for seven non-canonical intronic variants in *COL4A5* (6 reported variants and one from our cohort) by using *in vitro* splicing analysis with or without *in vivo* RNA sequencing. Consequently, five variants were expected to cause aberrant splicing (by skipping the respective exon) while one variant was found less likely to alter the splicing pattern (15). From the above, we should carefully judge the pathogenicity of non-canonical intronic variants and transcript analysis is recommended to assess their influence on splicing.

It has been known that deep intronic variants in *COL4A*5 also can cause aberrant splicing. Although this type of variant can be confirmed by only mRNA analysis because vast majority of intronic substitutions are polymorphisms, several pathogenic variants in deep introns of *COL4A5* have been reported (14, 25). While variants close to exon-intron boundary frequently cause exon skipping, deep intronic variants in *COL4A5* show the pathogenicity by the creation of cryptic exon (26, 27).

## Diagnostic Strategy of *COL4A5* Splicing Variants

### Genetic Analysis for XLAS

Previously, Sanger sequencing was widely used for the genetic diagnosis of Alport syndrome. However, screening of all three Alport genes (*COL4A3/COL4A4/COL4A5*) by conventional Sanger sequencing is time-consuming because each gene contains ~50 exons with no hotspots. Therefore, targeted exome sequencing with Next Generation Sequencing (NGS) has become the first line screening method for the genetic analysis. However, it should be noted that targeted exome sequencing, which screen exons and exon–intron boundaries, cannot detect all types of variant.

For example, large deletion across exons and copy number variations (CNVs) could not be screened by direct sequence and multiplex ligation-dependent probe amplification (MLPA) is the only way to detect this type of variant. However, a paired analysis approach, comparing NGS data of patients and normal controls, recently enabled us to screen CNVs and we detected *COL4A5* CNVs successfully with this method (28). Similarly, deep intronic variants causing aberrant splicing also could not be detected by standard sequencing for exons and exon-intron boundaries. Although whole genome sequencing (WGS) approach can screen deep intronic variants, it is difficult to detect pathogenic variants located in introns among the vast majority of non-pathogenic polymorphisms. Therefore, it is essential to carry out transcriptional analysis such as RNA sequencing to detect deep intronic variants.

Transcriptional analysis is important to assess the pathogenicity of intronic variants located outside canonical dinucleotides splice site (GU/AG). Variants located in canonical dinucleotide splice site can be diagnosed as pathogenic because of its critical effect on splicing (29), other intronic variants need to be assessed by transcriptional analysis to determine whether they cause aberrant splicing or not. In addition, it should be noted that there is a rare exception (<1%) of the type of canonical dinucleotide splice site (i.e., GC/AG etc.) and variants in this site do not always lead to exon skipping but sometimes partial deletion of exons or exonization of introns, which is important information to estimate renal prognosis for the evaluation of in-frame or out-of-frame deletions at the transcript levels (11, 14).

**Figure 2:**
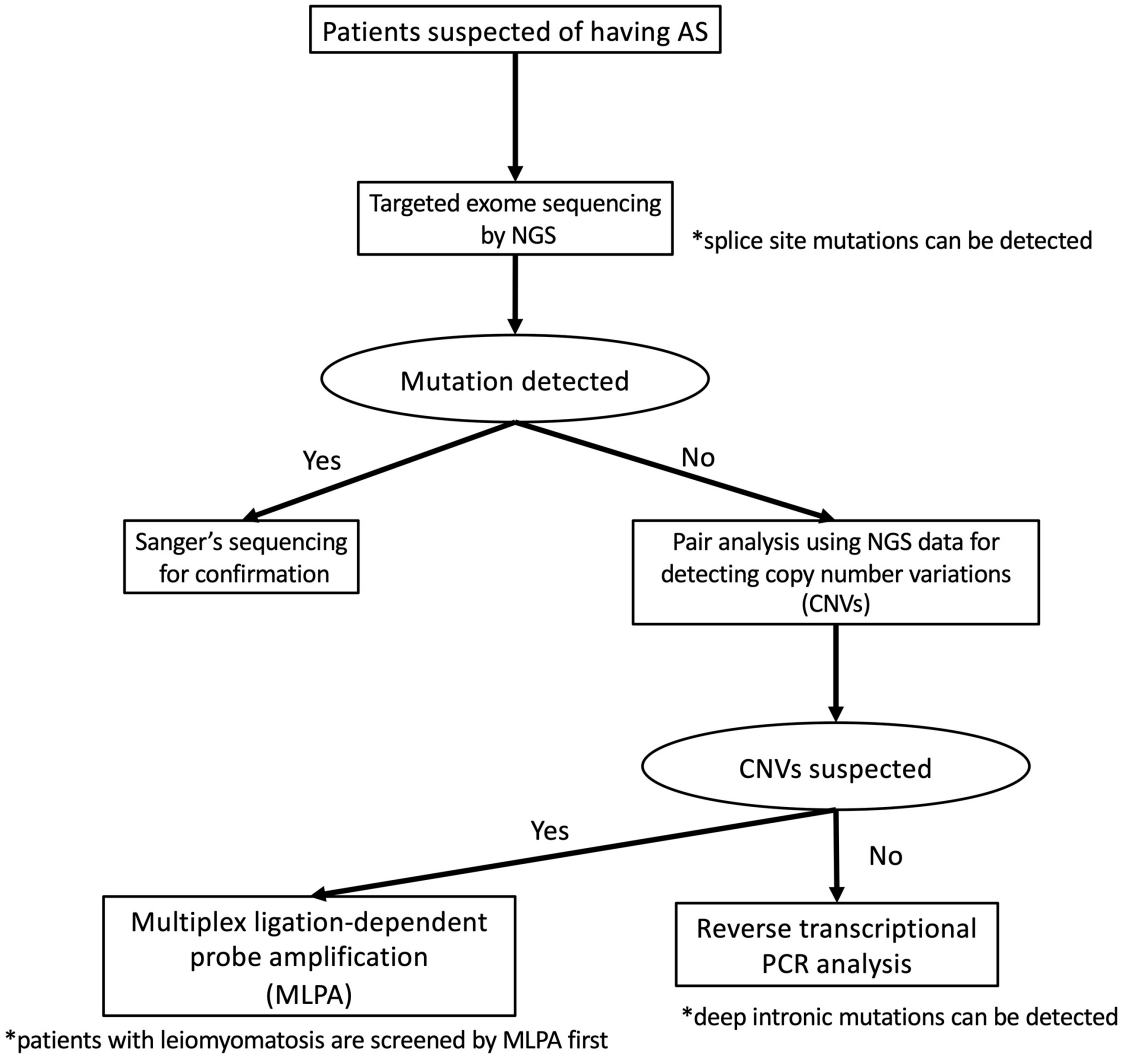
Mutational analysis approach for patients suspected as having Alport syndrome in our laboratory. In our laboratory, patients suspected as having Alport syndrome are first screened targeted exome sequencing by using NGS. If this first screening does not detect any pathogenic variants, screening for copy number variations (CNVs) in the *COL4A4/COL4A4/COL4A5* genes is performed using paired analysis. When paired analysis detects the possibility of CNVs in cases, multiplex ligation-dependent probe amplification (MLPA) is used to confirm. In addition, for patients with no obvious pathogenic variants, RNA sequencing using reverse transcription PCR is performed.

To detect pathogenic variants including splicing variants effectively, we conduct the genetic analysis for patients suspected as having XLAS in a stepwise manner, as shown in Figure 2. Briefly, targeted exome sequencing including all three Alport genes is performed first and then screening for copy number variations (CNVs) in the *COL4A3/ COL4A4/COL4A5* genes is performed using paired analysis for patients in whom no pathogenic variants are detected by targeted exome sequencing. When paired analysis detects the possibility of CNVs in cases, MLPA is used to confirm CNVs. In addition, for patients with no obvious pathogenic variants, RNA sequencing using RT-PCR will be performed to detect aberrant splicing by intronic variants.

Although the frequency of patients with CNVs is lower than those with splicing variants, our screening for CNVs can be conducted using the data of NGS analysis therefore this step is placed earlier than RNA sequencing. In addition, as the screening for CNVs using NGS data may not sufficiently detect CNVs of small size (smaller than 1,000 bp), we add MLPA analysis for the patients who are strongly suspected of having Alport syndrome from their clinical findings even if any pathogenic variants in *COL4A3/COL4A4/COL4A5* genes were not detected by NGS and RNA sequencing.

### Source of Transcriptional Analysis (Blood, Kidney, Hair Root, and Urine Derived Cell)

Peripheral leukocytes or hair roots have been traditionally used as a common source of mRNA for *COL4A5* transcriptional analysis because of its accessibility. However, the expression level of *COL4A5* mRNA in these samples is low and the nested PCR technique is required to amplify the targeted regions (14, 30). Although mRNA from patient kidney biopsies has abundant *COL4A5* expression, the cDNA analysis for *COL4A5* by using mRNA from kidney is not a common procedure since we now conduct a genetic analysis first approach for the diagnosis of Alport syndrome without performing a kidney biopsy. In contrast, patient urine samples have good accessibility and mRNA directly extracted from urine sediments is also abundant in *COL4A5* expression and can be used for RT-PCR (10, 11, 14, 23).

Urine-derived cells have been shown to express podocyte markers, and podocytes are the main source of type IV collagen α3α4α5 in the glomerular basement membrane (31, 32). Sergio et al. reported that urine derived “podocyte-lineage” cells from a patient with Alport syndrome expressed all three Alport gene mRNA and could be used for RT PCR analysis (32). Our group also use this cultured urine derived cells as a main source of mRNA for RNA sequencing of Alport genes. Comparing to direct extraction of mRNA from urine sediment, mRNA from urine derived cultured cells is easy to handle because of its larger and stable amount of RNA.

### *In vitro* Splicing Assay (Minigene Analysis)

**Figure 3:**
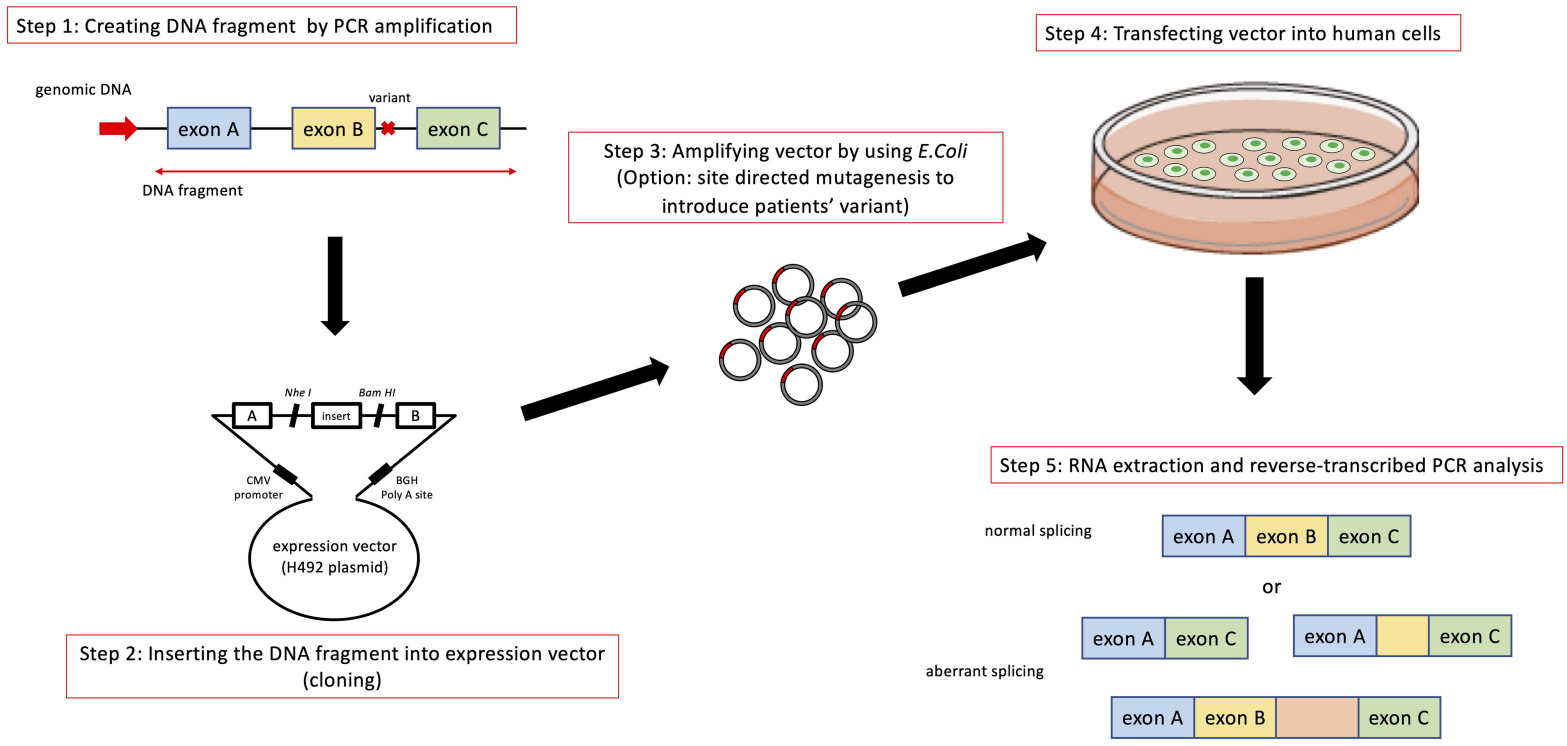
Schematic diagram of minigene analysis to test possible splicing variants. Step 1: Cleaving DNA fragment including the position of mutation by PCR from wild type and patient's gDNA. In addition to the exon close to the mutation being analyzed, we routinely include prier and following exons in DNA fragment. Step 2: Hybrid minigene constructs are created by inserting a test sequence fragment consisting of target exons and its flanking introns into the multicloning site within an intervening intron between two exons (exon A and B) of the minigene construct. Step 3: The hybrid minigene (cloning reaction) is amplified using *E. coli*. If patient's gDNA is not available, we introduce test mutation by using mutagenesis PCR. Step 4 and 5: These hybrid minigenes (both wild type and mutant) are transfected into human derived cells and culture them to express minigene derived mRNA. Step 5: Total RNA is reverse-transcribed into cDNA and the PCR is performed using specific. PCR products are analyzed by means of electrophoresis and direct sequencing.

In addition to transcript analysis using mRNA from patient derived samples, an *in vitro* functional splicing assay (minigene analysis) using an expression vector is also useful and can be applied for possible splicing variants in *COL4A5*. We routinely examine novel intronic variants or variants suspected of causing aberrant splicing by using a minigene assay, which is constructed to encode two cassette exons (A and B), an intervening sequence containing a multiple cloning site and a promoter region (Figure 3).

The analytical method of this assay is shown in Figure 3. Briefly, hybrid minigene constructs are created by inserting a test sequence fragment consisting of target gDNA region (exons) and the flanking introns into the multiple cloning site within an intervening intron between two exons (exon A and B) of the minigene construct. If a patient sample (or gDNA) is not available, the variant is introduced by site-directed mutagenesis using PCR. Then, hybrid minigenes are transfected into cultured human cells and total RNA was reverse- transcribed into cDNA and the PCR will be performed. Finally, PCR products are analyzed by means of electrophoresis and direct sequencing (Figure 3).

As described earlier, it is often hard to obtain samples for transcript extraction with high expression of target genes for inherited kidney diseases. However, the minigene assay has high flexibility because it does not require any mRNA or even gDNA samples from patients. Variants can be introduced to the minigene by using site directed mutagenesis allowing transcript analysis even if patient sample is not available (33, 34). We have analyzed several novel intronic variants in various inherited kidney diseases using this assay (35–40). As for intronic variants of *COL4A5*, several studies report using the minigene assay including our studies (11, 15, 41). In addition, most recently, our group applied this *in vitro* splicing assay for variants located in exons of *COL4A5* to clarify the characteristic of exonic splicing variants (22, 24).

## Therapeutic Approaches for Targeting Splicing Variants in *COL4A5*

As described elsewhere (42, 43), there are currently no curative therapies for Alport syndrome including XLAS. Current standard of care is with nephroprotective drugs such as renin-angiotensin-aldosterone system (RAS) blockade. Additional agents including bardoxolone methyl, the mi-RNA 21 inhibitor, endothelin receptor blocker, and sodium-glucose transport protein 2 (SGLT2) inhibitors are being trialed in Alport syndrome. Therapy is important and there is a difference in the treatment effect of RAS blockade for male XLAS patients between genotypes, truncating and non-truncating variants (10). However, non-specific nephroprotective therapy is currently not enough to prevent ESKD in their early age for the patients with truncating variants and the development of disease targeted new therapy is required.

As an inherited disease, the causative gene *COL4A5* can be a target of future therapy in addition to non-specific kidney protective therapies. Although one potential gene therapy is direct editing of variants by CRISPR-Cas based system or supplying complete *COL4A5* cDNA by using AAV vector, these approaches have not been successfully applied to XLAS so far. In addition, the process of splicing also attracts considerable attention as its potential therapeutic target in various inherited diseases in recent years. Specifically, antisense oligonucleotides (ASOs) therapy has been approved for several inherited diseases such as spinal muscular atrophy (SMA) and Duchene muscular dystrophy (DMD) and its application for Alport syndrome is also expected (44–49).

Our laboratory has already developed the ASO-mediated exon skipping therapy for the male XLAS model mice (with specific *Col4a5* nonsense variant) and reported its effects in 2020 (50). As described earlier, male XLAS patients have strong genotype-phenotype correlation that patients with truncating variants show severer kidney phenotype than those with non-truncating variants including in-frame deletion variants even at the transcript level. We aimed to amend the truncating transcript caused by the nonsense variant in *COL4A5* to an in-frame transcript by using ASO. Our ASO was designed to combine to the splicing regulatory motif on *COL4A5* pre-mRNA and to introduce exon skipping. As a result, we successfully proved its treatment effectiveness in XLAS model mice treated with ASO by showing a significant improvement compared to vehicle treated mice both clinically and pathologically (50).

Exon skipping therapy using ASO is potentially applied for some of splicing variants in *COL4A5*. About half of these variants result in truncating transcript due to skipping of exon where the nucleotide number is not a multiple of three (out-of-frame deletion). For these variants, additional exon(s) skipping introduced by ASO may be applied as treatment. If the total nucleotide number of skipped exons is a multiple of three, final transcript is in-frame and may lead milder phenotypes. This multiple exon skipping approach has been already considered as a promising treatment for DMD (51–53). In addition, splicing variants which lead to cryptic exon activation are a potential target of this exon skipping approach. A cryptic exon skipped transcript is normal and would result in a much milder phenotype.

In addition to ASO, cell-permeable RNA-targeting small molecules are also attract attention as novel candidates of splicing modifying treatment. A part of the small molecules binds RNA and has an influence on the splicing process. Most recently, risdiplam was approved by FDA as the first small molecule splicing modifier for SMA (54). It was shown that risdiplam analogs directly bind to pre-mRNA of SMN2 to introduce the inclusion of exon 7, which is usually skipped, thereby restoring the production of the SMN2 protein (55). This exon inclusion approach by a small molecule has also been tried for familial dysautonomia caused by intronic variants (IVS20 + 6T>C) in *IKBKAP* resulting in exon 20 skipping. Two different small molecules have also been reported to increase the normal splicing with inclusion of exon 20 in patients or patient derived cells (56, 57). Small molecules have advantages in terms of the administration route-ASOs are typically administered via intravenous, percutaneous or intrathecal routes, whereas small molecules can be administered orally.

Major problems of developing splicing modifying ASOs or small molecules for XLAS are the specificity of mechanism of action of these drugs and rarity of the treatment-amenable patient population. Each ASO or small molecule can bind a specific region of RNA and shows the action of splicing regulator and one drug may applicable for a few variants. As shown in previously, XLAS has no hotspot region and drugs therefore need to individual exons. However, there has been much interest in the development of individualized treatments for inherited diseases with small numbers of patients, and the FDA has issued a recommendation for the development of individualized ASOs to facilitate progress to individualized therapy.

## Conclusion

Splicing variants account for significant proportion of the total variants in XLAS and this proportion might be increasing accompany with advances of gene analysis in future. Thus, it is recommended to conduct RNA sequencing for the patients suspected as having XLAS in whom standard exome sequencing did not detect any variants (6). In addition, even for the cases with obvious splicing variants caused by canonical dinucleotides splice site (GU/AG) variants, transcript analysis by RNA sequencing or *in vitro* functional splicing assays will clarify the transcript pattern. This is important due to the difference in kidney prognosis of male XLAS patients between transcript types (in-frame vs. out-of-frame). Moreover, it should be noted that even exonic nucleotide substitutions can cause aberrant splicing and we should consider transcript analysis of those who have variants in specific regions (end of each exons) or show an atypical phenotype for their genotype; patients with severe phenotypes accompanied by missense variants or mild phenotype by nonsense mutation. Development of novel disease specific therapy targeting splicing mechanisms for XLAS is expected in the future.

## Author Contributions

TH wrote the Section of intronic variants outside the canonical splice site causing abnormal splicing in *COL4A5*. YA wrote the Section of exonic variants causing abnormal splicing in *COL4A5*. TY wrote the remaining sections. RL and KN critically reviewed the manuscript. All authors read and approved the final version of the manuscript.

## Funding

This work was supported by grant from the Uehara Memorial Foundation (No. 202040073 to TY).

## Conflict of Interest

The authors declare that the research was conducted in the absence of any commercial or financial relationships that could be construed as a potential conflict of interest.

## Publisher's Note

All claims expressed in this article are solely those of the authors and do not necessarily represent those of their affiliated organizations, or those of the publisher, the editors and the reviewers. Any product that may be evaluated in this article, or claim that may be made by its manufacturer, is not guaranteed or endorsed by the publisher.
